# Evidence for functional improvement in reward anticipation in recent onset schizophrenia after one year of coordinated specialty care

**DOI:** 10.1017/S0033291722003592

**Published:** 2023-10

**Authors:** Jason Smucny, Tyler A. Lesh, Tara A. Niendam, J. Daniel Ragland, Laura M. Tully, Cameron S. Carter

**Affiliations:** Department of Psychiatry and Behavioral Sciences, University of California, Davis, Sacramento, CA 95817, USA

**Keywords:** Anterior cingulate, fMRI, insula, positive symptoms, ventral striatum

## Abstract

**Background:**

Motivational impairment associated with deficits in processing the anticipation of future reward is hypothesized to be a cardinal feature of schizophrenia spectrum disorders (SZ). Evidence from short-term follow-up (6-week post-treatment) studies suggests that these deficits may improve or be reversed with treatment, although longer-term outcomes are unknown. Here we examined the one-year trajectory of functional activation in brain circuitry associated with reward anticipation in people with recent onset SZ who participated in coordinated specialty care (CSC) treatment, hypothesizing normalization of brain response mirroring previous short-term findings in first-episode individuals.

**Method:**

Blood oxygen level-dependent (BOLD) response in the dorsal anterior cingulate cortex, anterior insula, and ventral striatum (VS) associated with reward anticipation during the Incentivized Control Engagement Task (ICE-T) was analyzed in a baseline sample of 49 healthy controls (HCs) and 52 demographically matched people with SZ, with follow-up data available for 35 HCs and 17 people with SZ.

**Results:**

In agreement with our hypothesis, significant time × diagnosis interactions were observed across all regions, in which reward anticipation-associated BOLD response increased in SZ to above baseline HC levels at follow-up. Increased VS activation was associated with decreased reality distortion symptoms over the follow-up period. Baseline reward anticipation-associated BOLD response in the right anterior insula was associated with improvement in reality distortion symptoms.

**Conclusions:**

These findings suggest that functional deficits in reward anticipation may be reversed after one year of CSC in recent onset participants with SZ, and that this improvement is associated with reduced positive symptoms in the illness.

## Introduction

Impairments in reward processing are widely documented in schizophrenia spectrum disorders (SZ). Early viewpoints suggested a relative inability of people with the illness to experience pleasure (Blanchard, Mueser, & Bellack, 1998; Meehl, 1962). Recent reconceptualization, however, suggests that these impairments are more accurately characterized as a problem in reward anticipation, with ‘in the moment’ reward processes intact (Gold, Waltz, Prentice, Morris, & Heerey, 2008; Strauss, Waltz, & Gold, 2014). This pathological state is posited to result from a reduced ability to process and maintain value representations of different choices as well as an impairment in rapid learning based on trial-to-trial feedback (Barch & Dowd, 2010; Gold et al., 2008; Morris, Quail, Griffiths, Green, & Balleine, 2015; Strauss et al., 2014; Waltz, Frank, Wiecki, & Gold, 2011; Waltz & Gold, 2007).

Accordingly, a considerable amount of research has examined the functional neuronal basis of deficits in reward anticipation in SZ. A 2015 meta-analysis of 23 functional magnetic resonance imaging (fMRI) studies found significantly decreased activation of the ventral striatum (VS) during reward anticipation in SZ (Radua et al., 2015). A more recent (2020) meta-analysis reported lower activation during reward anticipation in SZ of several brain areas, including the prefrontal cortex, dorsal and VS, and anterior cingulate (Leroy et al., 2020). Adding to this literature, in 2021 our group published an fMRI analysis of the Incentivized Cue Engagement Task (ICE-T), finding significantly reduced activation of the insula during reward anticipation in recent onset SZ (Smucny et al., 2021). VS activity was qualitatively (but not significantly) lower in SZ in this study as well.

A few previous functional neuroimaging studies have also used longitudinal designs to examine the short-term effects of antipsychotic treatment on reward anticipation in first-episode illness. Nielsen et al. (2012) found significantly reduced VS activity associated with reward anticipation during the monetary incentive delay task at baseline in first-episode, antipsychotic-naïve individuals with SZ that normalized after 6 weeks of antipsychotic treatment. This normalization also positively correlated with degree of improvement in positive symptoms after treatment. Normalization of reward anticipation-associated response after 6 weeks of antipsychotic treatment was later replicated in an independent cohort of first-episode SZ individuals (Wulff et al., 2020).

These previous findings suggest that, in the short term, functional deficits during reward anticipation may respond positively to treatment in recent onset SZ. It remains unclear, however, if a similar pattern of normalization occurs over a more extended period. In this study, we used fMRI to examine the functional neuronal correlates of reward anticipation in individuals with recent onset SZ at baseline and one-year follow-up. Results showing reduced reward anticipation-associated insula activation in SZ at the ‘baseline’ timepoint of this study has been reported previously (Smucny et al., 2021). Consistent with short term longitudinal findings, we hypothesized increased activation of brain regions associated with reward processing in SZ at follow-up. Exploratory correlations with change in symptoms over the follow-up period were also conducted.

## Methods

### Participants

Forty-nine healthy controls (HCs) and 52 individuals with recent-onset, DSM-IV SZ (including schizophrenia, schizoaffective disorder, schizophreniform disorder, and psychosis-not-otherwise-specified) were recruited from the UC Davis Early Psychosis Programs (Early Diagnosis and Preventative Treatment (EDAPT) and SacEDAPT Clinics) and included in cross-sectional, ‘baseline’ results published previously (Smucny et al., 2021). All individuals were between 14 and 35 years of age. SZ participants were within two years of their first psychotic episode.

As part of their treatment at EDAPT and SacEDAPT, all participants with SZ were actively enrolled in coordinated specialty care (CSC) from baseline to one-year follow-up (Heinssen, Goldstein, & Azrin, 2014). This program, specifically designed for people with recent onset or first-episode psychosis, utilizes a personalized treatment regimen that may involve psychotherapy and counseling, family education and support, and work/education support in addition to standard pharmacologic-based treatment.

### Clinical ratings

Psychopathology was assessed using the Structured Clinical Interview for DSM-IV Axis I Disorders (First, Spitzer, Gibbon, & Williams, 2002). Symptoms were assessed using the Scale for the Assessment of Negative Symptoms (SANS) (Andreasen, 1984a), Scale for the Assessment of Positive Symptoms (SAPS) (Andreasen, 1984b), and Brief Psychiatric Rating Scale (BPRS) (Ventura et al., 1993). Consistent with prior work (Barch, Carter, MacDonald, Braver, & Cohen, 2003) three core symptom dimensions were calculated. ‘Poverty’ combined emotional withdrawal, motor retardation, and blunted affect from the BPRS with anhedonia/asociality, avolition/apathy, alogia, and affective flattening from the SANS. ‘Disorganization’ combined conceptual disorganization, mannerisms and posturing, and disorientation scores from the BPRS with attention score from the SANS as well as positive formal thought disorder, and bizarre behavior scores from the SAPS. ‘Reality distortion’ combined grandiosity, suspiciousness, hallucinations, and unusual thought content from the BPRS with hallucinations and delusions from the SAPS (Barch et al., 2003). Participants were excluded for a diagnosis of major medical or neurological illness, head trauma, substance abuse in the previous 3 months and/or a positive urine drug screen on the day of scanning, Weschler Abbreviated Scale of Intelligence (WASI) (Weschler, 1999) score < 70, and MRI exclusion criteria (e.g. claustrophobia, metal in the body). HC participants were also excluded for a history of Axis I mental illness or first-degree family history of psychosis. Chlorpromazine equivalent antipsychotic doses were calculated using published guidelines for conventional (American Psychiatric Association, 1997) and atypical (Woods, 2003) antipsychotics.

### Task description

The ICE-T is described in detail in our previous cross-sectional study (Smucny et al., 2021). The ICE-T is a delayed match-to-sample task that dissociates reward motivation and top-down cognitive control (Ursu & Carter, 2005; Ursu, Clark, Stenger, & Carter, 2008). Briefly, the task was composed of blocks of ‘same’ trials requiring low cognitive control and blocks of ‘opposite’ trials requiring high cognitive control (online Supplementary Fig. S1). Participants were alerted to the type of block by the words ‘same’ or ‘opposite’ that appeared on the screen before each block. These blocks were further composed of ‘neutral’ or ‘rewarded’ trials. Individuals were alerted via a pre-stimulus cue if a trial was to be ‘neutral’ or ‘rewarded.’ Participants were given response buttons for each hand. Additional task details are provided in online Supplementary Methods and Supplementary Table S1.

As previously (Smucny et al., 2021), only individuals who showed at least 60% accuracy during all four task conditions were included in analyses.

### fMRI image acquisition & preprocessing

3T images were acquired on a Siemens TimTrio scanner with an 8-channel head coil using a gradient *T*_2_ × −weighted echo planar imaging (EPI) sequence. Images were preprocessed with Statistical Parametric Mapping-8 (SPM8) software (http://www.fil.ion.ucl.ac.uk/SPM8). Functional data were reoriented, slice-time corrected, and realigned. Functional runs were excluded if they exceeded 0.45 mm frame-wise displacement and the entire participant was excluded if this movement cutoff was exceeded on 4 or more task runs. Of the previously analyzed baseline sample that met these movement criteria (Smucny et al., 2021), no participants were excluded based on movement criteria at follow-up.

Additional details on acquisition and preprocessing are provided in online Supplementary Material.

### First-level analysis

First-level blood oxygen level-dependent (BOLD) effects were modeled using a double-gamma function without temporal derivatives in a mixed event-related design using the general linear model function in SPM8. A 75s high-pass filter was employed. Images were motion-corrected using rigid-body motion parameters as single-subject regressors. All trial types were modeled (Same Neutral, Same Reward, Opposite Neutral, Opposite Reward) and only correct responses were used to create first-level images of cue and feedback-associated BOLD response. The contrast of interest was Reward > Neutral during the cue phase (corresponding to reward anticipation-associated BOLD activation). As the Opposite > Same contrast (designed to index cognitive control-associated BOLD activation) did not recruit the expected prefrontal areas in our cross-sectional baseline study (Smucny et al., 2021), we did not examine this contrast longitudinally in the present study.

### Regions of interest

Regions of interest (ROIs) were the same as our previous ICE-T study (Smucny et al., 2021), which were based on a 2012 meta-analysis of high reward > no reward anticipation fMRI contrasts (Diekhof, Kaps, Falkai, & Gruber, 2012). Specifically, reward anticipation (cue)-associated activation was extracted from 4 mm radius spherical ROIs from the dorsal anterior cingulate cortex (ACC), right anterior insula, left VS, and right VS using the Marsbar toolbox (Brett, Anton, Valabregue, & Poline, 2002). ROI MNI coordinates were centered at *x* = 2, *y* = 30, *z* = 32 for the dorsal ACC, *x* = 38, *y* = 20, *z* = −8 for the right anterior insula, *x* = −10, *y* = 10, *z* = −2 for the left VS, and *x* = 12, *y* = 12, *z* = −4 for the right VS. Brain images showing ROI locations are provided in online Supplementary Fig. S2.

### Group analysis and clinical correlations

Age, education, and WASI IQ were compared between groups by *t* tests. Group differences in sex and handedness were assessed by chi-square tests. Significance for these tests was set to *p* < 0.05.

In the SZ group, effects of time on clinical information (medication and symptoms) were analyzed by either linear mixed models ANOVAs (in the case of continuous outcome data; analysis conducted using proc MIXED (SAS University Edition, Cary, NC)) or a categorical mixed model (for comparing the ratio of medicated to unmedicated patients across time; analysis conducted using proc GLIMMIX) with time as a repeated within-subjects factor. Linear mixed effects models were estimated using the maximum likelihood method with unstructured covariance due to fit (based on Akaike's and Bayesian information criteria) and/or convergence. The categorical mixed model used a simple diagonal covariance structure for reasons of model fit as described above.

Accuracy and reaction time data were analyzed by mixed model ANOVA with time as a repeated within-subjects factor, reward condition and cognitive control condition as within-subjects factors, and diagnosis as a between-subjects factor. For fMRI data, following single-subject BOLD signal extraction associated with the reward > neutral contrast within the ROIs, longitudinal group fMRI ROI analysis was performed by mixed model ANOVA with time (baseline *v.* follow-up) as a repeated within-subjects factor, ROI as a within-subjects factor, and group diagnosis (HC *v.* SZ) as a between-subjects factor. In addition to overall group effects and interactions, planned contrasts compared the effect of time on reward-anticipation associated activation between SZ and HC within each ROI. Behavioral and fMRI models were also estimated using the maximum likelihood method with unstructured covariance due to model fit.

fMRI movement data for the six rigid-body parameters (*x*/*y*/*z*/translation/rotation) were compared by mixed model ANOVA with time as a repeated within-subjects factor, movement direction as a within-subjects factor, and group as a between-subjects factor using the model parameters described above for fMRI ROI data.

For the above mixed model analyses, follow-up data were assumed missing at random. Although this assumption cannot be tested directly, to evaluate follow-up bias we compared demographic, task, and/or clinical (for the SZ group) data at baseline between people with and without follow-up data using two-tailed *t* tests with significance *p* < 0.05.

Exploratory analyses of change in reward anticipation-associated ROI BOLD responses between baseline and follow-up with changes in poverty, disorganization, and reality distortion symptoms were conducted using Pearson's *r* with significance *p* < 0.05. All data for these analyses were normally distributed. Similarly, we also performed exploratory tests of associations between baseline activation and change in symptoms as well as tests between change in BOLD response and change in antipsychotic medication dose.

## Results

### Demographic and clinical

The final sample in our previous cross-sectional, ‘baseline’ study (Smucny et al., 2021) included 49 HCs and 52 people with SZ. Of these individuals, 14 HCs and 35 persons with SZ were either lost to follow-up (*n* = 14 HC, 32 SZ) or did not meet task performance standards (see Methods; *n* = 3 SZ) at follow-up, leaving 35 HCs and 17 participants with SZ with both baseline and follow-up data. No significant effect of time, effect of diagnosis, or time × diagnosis interaction was observed on participant head movement during scanning (online Supplementary Table S2).

Demographic and clinical information for participants with data available for analysis is shown in Tables 1 and 2, respectively, with additional summary statistics excluding people lost to follow-up presented in online Supplementary Table S3. Groups did not differ by age, sex, handedness, or parental education. The SZ group had fewer years of education and lower WASI-2 IQ scores compared to HCs. Although education and IQ were different between groups, these measures were not controlled for in mixed effects models because they are associated with the clinical presentation of SZ. Thus, removing their effects would remove meaningful variance associated with the illness (Miller & Chapman, 2001). No group difference was observed in the number of days between baseline and follow-up. Mean days to follow-up were 360 (s.d. = 83) for HCs and 336 (s.d. = 43) for people with SZ.

**Table 1.** Demographic information for participants

	HC (s.d.)		SZ (s.d.)		Entire sample HC *v.* SZ *t* or χ^2^ (*p*)	People with complete data HC *v.* SZ *t* or χ^2^ (*p*)
	Entire sample	HCs with complete data (Baseline and follow-up)	Entire sample	SZs with complete data (Baseline and follow-up)		
*N*	49	35	52	17	–	–
*N* SZ/SZ-A/SZ-P/PNOS	–	–	38/10/3/1	12/3/2/0	–	–
Age	20.24 (3.00)	20.40 (3.23)	20.02 (3.82)	21.06 (4.16)	0.33 (0.74)	0.63 (0.53)
Sex M/F	33/16	24/11	39/13	13/4	0.72 (0.40)	0.35 (0.56)
Handedness L/R	2/46	2/33	4/48	3/14	0.55 (0.46)	1.88 (0.17)
Education level, Years	13.73 (2.56)	13.77 (2.61)	12.24 (2.01)	12.59 (1.77)	3.26 (0.002)	1.68 (0.10)
Parental education level, Years	14.98 (3.43)	15.30 (3.37)	14.81 (2.80)	14.64 (2.83)	0.26 (0.79)	0.68 (0.50)
WASI-2 IQ	118.61(12.55)	118.18 (13.46)	103.75 (15.41)	105.65 (17.80)	5.10 (<0.001)	2.79 (0.008)
Duration of illness, Days	–	–	274.22 (154.91)	288.94 (187.89)	–	–
Days between baseline and follow-up	360.14 (82.63)		335.71 (43.46)		1.40 (0.17)	

HC, healthy controls; PNOS, psychosis-not-otherwise-specified; s.d., standard deviation; SZ, schizophrenia spectrum disorder; SZ-A, schizoaffective disorder; SZ-P, schizophreniform disorder; WASI-2, Weschler Abbreviated Scale of Intelligence, 2nd Edition.

Handedness was unavailable for one HC. Education data were unavailable for 1 SZ participant. Parental education data were unavailable for 3 HC and 7 SZ participants. WASI scores were unavailable for 5 HC and 1 SZ participant(s). Illness duration data were unavailable for 1 SZ participant.

**Table 2.** Clinical information

	Baseline (s.d.)	1 Year (s.d.)	Baseline *v.* 1 Year *F* or *t* (*p*)
N Medicated/Unmedicated	47/5	12/5	*t* = 1.52 (0.13)
Antipsychotics CPZ equivalent dose, Mg/Day	196.5 (133.8)	219.9 (152.7)	*F* = 0.14 (0.71)
Poverty symptoms	15.06 (5.98)	10.56 (6.48)	*F* = 10.66 (0.003)
Disorganization symptoms	6.49 (2.97)	5.63 (2.39)	*F* = 0.62 (0.44)
Reality distortion symptoms	11.90 (7.30)	8.31 (5.68)	*F* = 2.81 (0.11)

CPZ, chlorpromazine; s.d., standard deviation; SZ, schizophrenia spectrum disorder.

Syndrome (poverty, disorganization, reality distortion) scores were unavailable for 3 people with SZ. Baseline data is taken from the entire sample (i.e. including people lost to follow-up).

Comparing clinical data between baseline and follow-up, a significant improvement was observed in poverty symptoms at follow-up (Table 2). No significant differences over time were observed for the ratio of medicated/unmedicated patients or antipsychotic dose.

### Behavioral analysis

Behavioral summary statistics and results are presented in Table 3, with additional summary statistics excluding people lost to follow-up presented in online Supplementary Table S3. For accuracy, a significant main effect of time, time × diagnosis interaction, and cognitive control × diagnosis interaction were observed. The time interaction effect was driven by larger decreases in accuracy in SZ at follow-up relative to baseline compared to HC. The cognitive control × diagnosis interaction effect was driven by a larger deficit in accuracy under the opposite condition relative to the same condition in SZ compared to HCs. No significant interactions between time and reward condition were observed, suggesting that reduced accuracy in SZ at follow-up was not dependent on reward.

**Table 3.** Task accuracies and reaction times for each group and trial type at each time point

	HC (s.d.)		SZ (s.d.)		*F* Time (*p*)	*F* Time × *D_x_* (*p*)	*F D_x_* × CC (*p*)	*F D_x_* × Rew × Time (*p*)
	Baseline (*n* = 49)	Follow-up (*n* = 35)	Baseline (*n* = 52)	Follow-up (*n* = 17)				
Accuracy								
Same neutral	0.92 (0.07)	0.91 (0.07)	0.89 (0.07)	0.85 (0.10)	14.94 (<0.001)	16.22 (<0.001)	8.31 (0.004)	2.61 (0.11)
Same reward	0.94 (0.05)	0.94 (0.06)	0.91 (0.07)	0.92 (0.07)				
Opposite neutral	0.85 (0.08)	0.85 (0.08)	0.82 (0.10)	0.76 (0.09)				
Opposite reward	0.89 (0.07)	0.88 (0.07)	0.85 (0.08)	0.81 (0.09)				
Reaction time ms								
Same neutral	551.8 (59.3)	544.6 (61.8)	562.2 (56.9	591.9 (69.4)	3.38 (0.067)	36.33 (<0.001))	1.69 (0.19)	3.92 (0.048)
Same reward	537.4 (62.4)	530.1 (58.3)	550.1 (53.9)	566.9 (70.1)				
Opposite neutral	596.1 (67.2)	585.6 (66.1)	610.1 (60.9)	641.0 (80.3)				
Opposite reward	583.3 (65.2)	577.7 (60.3)	608.4 (60.1)	616.6 (74.0)				

CC, cognitive control; HC, healthy controls; s.d., standard deviation; SZ, schizophrenia spectrum disorder.

All non-listed interactions were non-significant.

For reaction time, significant time × diagnosis and time × diagnosis × reward interactions were observed. These effects were driven by slower reaction times at follow-up *v.* baseline in the SZ group (particularly during the opposite neutral condition) but slightly faster reaction times in the HC group at follow-up.

### fMRI region of interest analysis

**Fig. 1.**
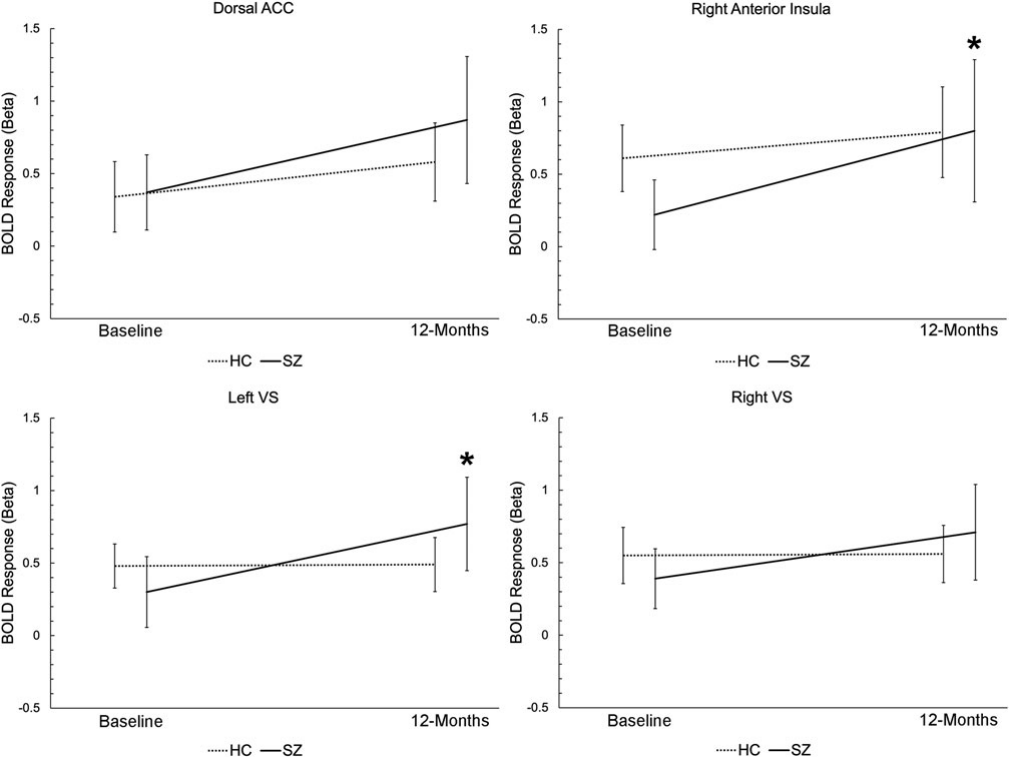
Reward anticipation-associated activation for each region of interest at baseline and 12-month follow-up for healthy controls (HCs) and people with schizophrenia spectrum disorders (SZ). ACC, anterior cingulate cortex; BOLD, blood oxygenation level-dependent; HC, healthy control; VS, ventral striatum. *p < 0.05 *v.* baseline.

fMRI ROI analysis results are presented in Fig. 1 and online Supplementary Table S4, with additional summary statistics excluding people lost to follow-up presented in online Supplementary Table S3. Across all ROIs, a significant main effect of time (*F* = 37.06, *p* < 0.001), time × diagnosis interaction (*F* = 9.60, *p* = 0.002), and diagnosis × region interaction (*F* = 2.87, *p* = 0.036) were observed. Planned contrasts within each ROI revealed the effect was driven by significantly greater increase in activation at follow-up (*v.* baseline) in SZ (*v.* HC) in the right anterior insula and left VS.

### Comparison of participants with *v.* without follow-up data

T-tests comparing demographic, task, and/or clinical (for the SZ group) data at baseline between people with *v.* without follow-up data are provided in online Supplementary Table S5. No significant differences were observed in demographic or clinical information or in reward anticipation-associated activation in any ROI. HCs with data lost to follow-up showed significantly higher accuracy during the same reward condition and showed lower y-translational scanner movement.

### Clinical correlations

In SZ, a significant negative association was observed between change in reward anticipation-associated activation at follow-up (*v.* baseline) in the left VS and change in reality distortion symptoms (*r* = −0.66, *p* = 0.008), suggesting that symptom improvement was associated with increased VS activity at follow-up. Baseline reward anticipation-associated activation in the right anterior insula was also positively correlated with improvement in reality distortion symptoms from baseline to follow-up (*r* = 0.53, *p* = 0.040). No other associations between change in reward anticipation-associated activation at follow-up (*v.* baseline) in ROIs and symptom domains were observed. No associations were observed between change in reward anticipation-associated functional activation of any ROI and change in antipsychotic dose over the follow-up period.

## Discussion

In agreement with our hypothesis, in this study people with recent onset SZ showed significantly increased functional activation associated with reward anticipation in two reward processing-associated brain regions, the anterior insula and VS, over the course of one-year of CSC relative to demographically matched HC participants. A negative association was also observed between change in left VS activity and reality distortion symptom score, suggesting that increased activation is correlated with improvement in positive symptoms during CSC. In addition, a positive association was observed between reward anticipation-associated activation in the right anterior insula and improvement in reality distortion symptoms, suggesting that activation in this region during the task may be a predictor of future clinical improvement. Changes in activation were also unrelated to change in antipsychotic dose, suggesting they are not influenced by change in neuroleptic chlorpromazine equivalents. No significant differences were noted in any baseline fMRI activation measure between people with and without follow-up data, suggesting results were not significantly biased based on the availability follow-up information.

In our previous, cross-sectional publication that examine reward anticipation-associated BOLD response at baseline, we observed significantly reduced activity in the right anterior insula in SZ as well as qualitatively lower activation in the right and left VS (Smucny et al., 2021). As mean activation in all four ROIs at follow-up in SZ was greater than baseline HC levels (online Supplementary Table S4), the results of the present study suggest that after one year of CSC, activation levels may approach (and even move beyond) HC levels. The fact that the diagnosis × time interaction was significant also suggests the improvement in SZ was not due to a non-SZ-related, generalized practice effect. The preliminary results presented here are conceptually consistent with the short term (6 week) longitudinal studies by Nielsen et al. (2012) and Wulff et al. (2020) who showed reversal of functional deficits in reward anticipation-associated activation in the caudate in first-episode individuals with SZ who undergo 6 weeks of antipsychotic treatment. Our results extend the scope of these previous findings by showing that functional reversal of reward anticipation deficits is apparent after a one-year period. It should be noted, however, that this was a naturalistic study that did not include a ‘placebo’ treatment group (i.e. one that did not undergo CSC). Furthermore, no associations were observed between change in antipsychotic dose and change in ROI response. We therefore cannot rule out the possibility that the observed improvement occurred due to natural disease progression or for other reasons unrelated to CSC.

In contrast to the pattern of functional results, patients did not show an improvement in behavioral performance (or time × reward interactions on performance) between baseline and follow-up. What factors may explain this discrepancy? First, performance levels were already quite high at baseline in the SZ group (Table 3), and the dynamic range for improvement was limited. Second, the observed improvement in function was specific to reward anticipation and does not necessarily suggest that other neuronal processes that may influence performance (e.g. motor networks, attention networks, or executive control networks) also improved over time in SZ. Thus, increased reward anticipation-associated activity may suggest improvement in motivation or arousal that may not necessarily ‘translate’ into observable behavioral effects. It is also possible that brain-behavior relationships are disrupted in SZ, such that changes in brain activation may be less associated with performance compared to healthy individuals (e.g. Avery et al., 2019).

Interestingly, exploratory analyses showed the observed increase in left VS activation was significantly associated with improvement in reality distortion symptoms in the SZ group. Similarly, in their work demonstrating normalization of caudate response after 6 weeks of antipsychotic treatment, Nielsen et al. (2012) and Wulff et al. (2020) also found that people with SZ that showed the most improvement in positive symptoms showed the greatest reversal in functional deficits. Associations between VS function during reward activation and positive symptoms are also consistent with a recent meta-analysis (Leroy et al., 2020). Heterogeneity in treatment response is a now well-studied phenomenon in the illness, possibly due stratification of striatal hyperdopaminergia, i.e. people who respond to antidopaminergic drugs may be more hyperdopaminergic at baseline (Kim et al., 2017; Veronese et al., 2021). Hyperdopaminergia and ‘noisy’ dopamine signals may also reduce phasic dopamine release during rewarding or otherwise relevant stimuli (with antipsychotic treatment reversing this effect of time), contributing to the observed functional pattern of findings (Maia & Frank, 2017). Related to this point, Wulff et al. (2020) also found that the degree of functional normalization correlated with D2 receptor occupancy as measured by single photon emission computed tomography imaging, linking these effects with dopaminergic blockade and suggesting that the people with SZ who were the most treatment responsive were mostly likely to show functional normalization. Although we did not see a relationship between antipsychotic dose and activation, it is possible that the observed increase in VS activity reflects drug efficacy in individual people with SZ due to baseline differences in striatal dopaminergic tone. It is also possible that non-pharmacologic effects of CSC, such as cognitive therapy, contributed to the observed pattern of findings. As most SZ participants were medicated at baseline in this study, future longitudinal studies comparing the longitudinal trajectories of reward anticipation-associated activation in antipsychotic naïve *v.* medicated CSC individuals may help shed light on these possibilities. Exploratory analyses also found a putative association between baseline reward anticipation-associated activation in the right anterior insula and improvement in reality distortion symptoms. One possible explanation for this association is that people with SZ with more intact reward anticipation-associated processing in this area are more motivated to adhere to treatment, enhancing effects of the multifaceted treatment regimen offered during CSC.

Our study had several limitations. First, the sample size for the SZ group at follow-up was small, as many individuals were lost to follow-up (i.e. dropped out of CSC or were excluded for data quality at baseline or follow-up). The findings reported here are therefore preliminary and would be strengthened by replication in an independent sample. Importantly, however, no baseline differences were found between demographic, clinical, or brain activation measures between individuals with and without follow-up data in either diagnostic group, suggesting the follow-up sample did not constitute a biased or otherwise categorically distinct sample from the initial baseline group. Second, given that generalized deterioration in cognition is generally not observed in recent onset SZ over the first few years of illness (Keefe, 2014; Niendam et al., 2018; Smucny et al., 2018, 2020), we unexpectedly found that people with SZ performed generally worse (i.e. were less accurate and slower) during the task at follow-up *v.* baseline. The limitation may be mitigated by the fact that although significant, the reduction in accuracy was quantitatively small (~5% across conditions). Furthermore, the reduction in accuracy occurred across task conditions and was not specific to reward. Finally, it is important to note that the patient sample in this study had been undergoing treatment for ~300 days on average (Table 1) prior to their baseline scanning. It is therefore possible that the observed effects were additive and/or represented a delayed effect from prior CSC treatment. It is also possible that patients at baseline were already ‘improved’ relative to what their activation levels would be if they were measured at first-episode.

In conclusion, the results of this preliminary study suggest that functional deficits in reward anticipation-associated activation may be reversed after one year of CSC in recent onset illness. These results extend those of previous short-term (6 week) longitudinal studies by showing that reversal effects may persist for at least one year. Future studies that compare functional trajectories between antipsychotic naïve *v.* medicated individuals will help determine if these effects are the result of neuroleptic treatment.

## Supplementary material

For supplementary material accompanying this paper visit https://doi.org/10.1017/S0033291722003592. click here to view supplementary material
