# Supplementary material for: Evidence for functional improvement in reward anticipation in recent onset schizophrenia after one year of coordinated specialty care
Source: Psychol Med. 2022 Nov 24;53(13):6280–7. doi: 10.1017/S0033291722003592 (PMC10520583; doi:10.1017/S0033291722003592)
Supplement: Supplementary file 1 [file S0033291722003592sup001.docx]

**Supplementary Methods**

*Task Description*

The ICE-T is a delayed match-to-sample task that dissociates reward motivation and top-down cognitive control (Ursu & Carter, 2005; Ursu, Clark, Stenger, & Carter, 2008). Briefly, the task was composed of blocks of "same" trials requiring low cognitive control and blocks of "opposite" trials requiring high cognitive control (Supplementary Figure 1). Participants were alerted to the type of block by the words "same" or "opposite" that appeared on the screen before each block. These blocks were further composed of "neutral" or "rewarded" trials. Individuals were alerted via a prestimulus cue if a trial was to be "neutral" or "rewarded." Participants were given response buttons for each hand.

ICE-T parameters are provided in Supplementary Table 1. Specifically, in ICE-T trials a cue stimulus first appeared on the screen for 0.5s. The color of the stimulus alerted the participant if the trial will be neutral or rewarded. Stimulus color mapping (i.e. if red or green corresponded to neutral or rewarded trials) and block order (same or opposite) were counterbalanced across participants. Following a 2 or 4s interstimulus interval, two side-by-side probe stimuli were then presented for 1s. One of the probe stimuli matched the previous stimulus and the other did not. In "same" (low cognitive control) blocks, the participant was instructed to press the button on the ipsilateral side of the matching stimulus, e.g., left hand button if the matching stimulus was on the *left* side. In "opposite" (high cognitive control) blocks, the participant was instructed to press the button on the contralateral side of the matching stimulus, e.g., left hand button if the matching stimulus was on the *right* side. 0.5 duration visual feedback was then provided. Specifically, in neutral trials, "OK" was displayed if the participant answered accurately within the time limit, and "X" was displayed otherwise. In rewarded trials, "$$" was displayed if the participant answered accurately within the time limit (earning $0.50), and "X" was displayed otherwise. Each trial was followed by a jittered intertrial interval of 4s on average, with minimum 2s and maximum 16s. Incentive condition (neutral or rewarded) and target location (left or right) was pseudorandomized and consequently counterbalanced within blocks. Each participant was presented with 6 runs of 6m 4s each, with each run consisting of 1 opposite block of 20 trials and 1 same block of 20 trials. The task therefore included 60 trials of each condition (Same Neutral, Same Reward, Opposite Neutral, Opposite Reward). Total task length was 36m 48s.

The initial response time limit of 1s was shortened by 10% after streaks of 5 correct and fast responses to rewarded trials. The initial response time limit of 1s was shortened by 10% after streaks of 5 correct and fast responses. Conversely, two consecutive errors, late responses, or null responses increased the response time limit by 10%. This adjustment was implemented to reduce changes in the reward expectation of each trial as the task went on (Ursu & Carter, 2005; Ursu et al., 2008). In addition, the adjustment may help minimize performance differences that might change the value of opposite vs. same trials independent of the reward status of the trial, because trials more likely to be answered correctly may induce more rewards for answering correctly (Ursu & Carter, 2005; Ursu et al., 2008).

Accuracy scores were calculated as the mean percent correct in response to the probe over all blocks of trials for each condition (Same Neutral, Same Reward, Opposite Neutral, Opposite Reward). Reaction time was calculated as the mean reaction time in response to correct probes over all blocks of trials for each condition.

*fMRI Image Acquisition & Preprocessing*

3T images were acquired on a Siemens TimTrio scanner with an 8-channel head coil using a gradient T2*-weighted echo planar imaging (EPI) sequence (32 interleaved axial-oblique slices; 3.8mm x 3.8mm x 3.5mm voxels; 75^∘^ flip angle; 2000ms TR; 29ms TE; 240 mm x 240mm field of view). BOLD images were aligned to the anterior commissure — posterior commissure + 10^∘^. High resolution anatomical images were also acquired for each participant using a 3-dimensional T1-weighted magnetization-prepared rapid acquisition of gradient-echo (MPRAGE) sequence (176 contiguous anterior commissure — posterior commissure slices; acceleration factor of 2; 1mm x 1mm x 1mm voxels; 7^∘^ flip angle; 2530ms TR; 3.5ms TE; 256 mm x 256mm field of view). Head movement was minimized using foam padding and participants wore earplugs to muffle scanner noise.

Images were preprocessed with Statistical Parametric Mapping-8 software (SPM8) (http://www.fil.ion.ucl.ac.uk/SPM8). Functional data were reoriented, slice-time corrected, and realigned. Structural images were brain-extracted using FSL’s BET toolbox (Smith, 2002) and then segmented and co-registered to the mean functional image. Images were spatially normalized to the Montreal Neurological Institute (MNI) template using a non-linear 12-parameter affine transformation and then smoothed with an 8mm kernel. Data were screened for image artifacts, structural abnormalities, and normalization errors to ensure data quality. Functional runs were excluded if they exceeded 0.45mm frame-wise displacement and the entire participant was excluded if this movement cutoff was exceeded on 4 or more task runs.

**Supplementary Table 1.** Task parameters. Abbreviations: ISI = Interstimulus Interval, ITI = Intertrial Interval.

| **Total Trials** | **Cue Duration ms** | **Probe Duration ms** | **Feedback Duration ms** | **ISI ms** | **ITI ms** | **Total Time** |
| --- | --- | --- | --- | --- | --- | --- |
| 6 blocks x 2 conditions (same or opposite) x 20 trials = 240 | 500 | 1000 | 500 | 2000 or 4000 | average 4000, range 2000 -14000, step 2000 (jittered) | 36m 48s |

**Supplementary Table 2.** Functional magnetic resonance imaging (fMRI) average movement data. Abbreviations: SD = Standard Deviation, SZ = Schizophrenia Spectrum Disorder.

| **Measure** | **HC (SD)** | | **SZ (SD)** | | | **F Time (*p*)** | | **F Dx (*p*)** | **F Time*Dx (*p*)** |
| --- | --- | --- | --- | --- | --- | --- | --- | --- | --- |
|  | **Baseline (*n* = 49)** | **Follow-Up (*n* = 35)** | **Baseline**  **(*n* = 52)** | **Follow-Up**  **(*n* = 17)** |  | |  | |  |
| **X-Translation mm** | .16 (.19) | .14 (.11) | .16 (.12) | .18 (.14) | .01 (.90) | | 2.40 (.12) | | .03 (.87) |
| **Y-Translation mm** | .15 (.09) | .17 (.09) | .18 (.09) | .20 (.10) |  |  |  |  |  |
| **Z-Translation mm** | .39 (.31) | .45 (.37) | .63 (.75) | .57 (.36) |  |  |  |  |  |
| **X-Rotation mm** | .32 (.31) | .32 (.29) | .32 (.29) | .33 (.17) |  |  |  |  |  |
| **Y-Rotation mm** | .13 (.08) | .12 (.05) | .13 (.10) | .15 (.05) |  |  |  |  |  |
| **Z-Rotation mm** | .13 (.19) | .11 (.10) | .12 (.09) | .13 (.07) |  |  |  |  |  |

**Supplementary Table 3.** Summary statistics (baseline and follow-up) for people with complete datasets, i.e., excluding participants lost to follow-up. Syndrome (poverty, disorganization, reality distortion) scores were unavailable for 3 people with SZ. Numbers in parentheses represent the standard deviation unless otherwise specified. **p* < 0.05. Abbreviations: ACC = Anterior Cingulate, CPZ = Chlorpromazine, HC = Healthy Control, SD = Standard Deviation, SE = Standard Error, SZ = Schizophrenia Spectrum Disorder, VS = Ventral Striatum.

|  | **HC (*n* = 35)** | | **SZ (*n* = 17)** | |
| --- | --- | --- | --- | --- |
|  | **Baseline** | **Follow-Up** | **Baseline** | **Follow-Up** |
| ***N* Medicated/Unmedicated** | — | — | 16/1 | 12/5 |
| **CPZ Equivalent Dose, Mg/Day** | — | — | 227.71 (191.61) | 219.9 (152.7) |
| **Poverty Symptoms** | — | — | 15.13 (4.21) | 10.56 (6.48) |
| **Disorganization Symptoms** | — | — | 5.94 (2.64) | 5.63 (2.39) |
| **Reality Distortion Symptoms** | — | — | 12.00 (5.91) | 8.31 (5.68) |
| **Accuracy** |  |  |  |  |
| Same Neutral | .91 (.07) | .91 (.07) | .92 (.06) | .85 (.10) |
| Same Reward | .93 (.05) | .94 (.06) | .92 (.06) | .92 (.07) |
| Opposite Neutral | .84 (.09) | .85 (.08) | .81 (.09) | .76 (.09) |
| Opposite Reward | .89 (.08) | .88 (.07) | .86 (.06) | .81 (.09) |
| **Reaction Time ms** |  |  |  |  |
| Same Neutral | 556.84 (63.61) | 544.6 (61.8) | 551.74 (55.81) | 591.9 (69.4) |
| Same Reward | 543.62 (69.03) | 530.1 (58.3) | 541.24 (55.3) | 566.9 (70.1) |
| Opposite Neutral | 602.47 (72.10) | 585.6 (66.1) | 602.99 (51.89) | 641.0 (80.3) |
| Opposite Reward | 588.87 (70.00) | 577.7 (60.3) | 599.30 (63.21) | 616.6 (74.0) |
| **fMRI Movement** |  |  |  |  |
| X-Translation mm | .16 (.21) | .14 (.11) | .14 (.11) | .18 (.14) |
| Y-Translation mm | .17 (.10) | .17 (.09) | .16 (.10) | .20 (.10) |
| Z-Translation mm | .40 (.33) | .45 (.37) | .47 (.28) | .57 (.36) |
| X-Rotation mm | .35 (.35) | .32 (.29) | .28 (.28) | .33 (.17) |
| Y-Rotation mm | .12 (.07) | .12 (.05) | .13 (.12) | .15 (.05) |
| Z-Rotation mm | .13 (.22) | .11 (.10) | .11 (09) | .13 (.07) |
| **Activation in ROIs** |  |  |  |  |
| Dorsal ACC | .33 (.78) | .58 (.78) | .46 (.59) | .87 (.84) |
| Right Anterior Insula | .54 (.83) | .79 (.90) | .21 (.79) | .80 (.96) |
| Left VS | .42 (.52) | .49 (.55) | .32 (.56) | .77 (.63) |
| Right VS | .50 (.69) | .56 (.57) | .33 (.41) | .71 (.64) |

|  | **HC (SD)** | | **SZ (SD)** | | | **Estimate, SZ>HC, Follow-Up > Baseline Mean Difference (SE)** | **Estimate t (*p*)** |
| --- | --- | --- | --- | --- | --- | --- | --- |
|  | **Baseline (*n* = 49)** | **Follow-Up (*n* = 35)** | **Baseline (*n* = 52)** | **Follow-Up (*n* = 17)** | |  |  |
| Dorsal ACC | .34 (.84) | .58 (.78) | .37 (.93) | | .87 (.84) | .20 (.18) | 1.13 (.26) |
| Right Anterior Insula | .61 (.82) | .79 (.90) | .22 (.86) | | .80 (.96) | .36 (.047) | 1.99 (.047) |
| Left VS | .48 (.54) | .49 (.55) | .30 (.87) | | .77 (.63) | .42 (.18) | 2.31 (.021) |
| Right VS | .55 (.67) | .56 (.57) | .39 (.73) | | .71 (.64) | .26 (.18) | 1.44 (.15) |

**Supplementary Table 4.** Adjusted beta estimates of BOLD response associated with reward anticipation within brain regions of interest (ROIs) for each group and time point. Abbreviations: ACC = Anterior Cingulate, HC = Healthy Control, SD = Standard Deviation, SE = Standard Error, SZ = Schizophrenia Spectrum Disorder, VS = Ventral Striatum.

**Supplementary Table 5.** Comparison of baseline data for individuals with vs. without follow-up data. Handedness was unavailable for one HC. Education data were unavailable for 1 SZ participant. Parental education data were unavailable for 3 HC and 7 SZ participants. WASI scores were unavailable for 5 HC and 1 SZ participant(s). Illness duration data were unavailable for 1 SZ participant. Syndrome (poverty, disorganization, reality distortion) scores were unavailable for 3 people with SZ. Numbers in parentheses represent the standard deviation unless otherwise specified. **p* < 0.05. Abbreviations: CPZ = Chlorpromazine, HC = Healthy Controls, PNOS=Psychosis-Not-Otherwise-Specified, SZ = Schizophrenia Spectrum Disorder, SZ-A = Schizoaffective Disorder, SZ-P = Schizophreniform Disorder, WASI-2 = Weschler Abbreviated Scale of Intelligence, 2nd Edition.

|  | **HC (*n* = 49)** | | **t or ^2^ (*p)*** | **SZ (*n* = 52)** | | **t or ^2^ (*p)*** |
| --- | --- | --- | --- | --- | --- | --- |
|  | **Complete (*n* = 35)** | **Lost to Follow-Up (*n* = 14)** |  | **Complete (*n* = 17)** | **Lost to Follow-Up (*n* = 35)** |  |
| ***N* SZ/SZ-A/SZ-P/PNOS** | — | — | — | 12/3/2/0 | 26/7/1/1 | 2.11 (.55) |
| **Age** | 20.40 (3.23) | 19.86 (2.41) | .57 (.57) | 21.06 (4.16) | 19.51 (3.59) | 1.38 (.17) |
| **Sex M/F** | 24/11 | 9/5 | .08 (.77) | 13/4 | 26/9 | .03 (.86) |
| **Handedness L/R** | 2/33 | 0/13 | 3.30 (.19) | 3/14 | 1/34 | 3.53 (.060) |
| **Education Level, Years** | 13.77 (2.61) | 13.64 (2.53) | .16 (.88) | 12.59 (1.77) | 12.06 (2.12) | .89 (.38) |
| **Parental Education Level, Years** | 15.30 (3.37) | 14.15 (3.58) | 1.02 (.31) | 14.64 (2.83) | 14.90 (2.82) | .29 (.77) |
| **WASI IQ** | 118.18 (13.46) | 119.91 (9.78) | .39 (.70) | 105.65 (17.80) | 102.79 (14.27) | .62 (.54) |
| **Duration of Illness, Days** | — | — | — | 288.94 (187.89) | 266.85 (138.11) | .48 (.64) |
| ***N* Medicated/Unmedicated** | — | — | — | 16/1 | 31/4 | .41 (.53) |
| **CPZ Equivalent Dose, Mg/Day** | — | — | — | 227.71 (191.61) | 179.92 (89.34) | .94 (.36) |
| **Poverty Symptoms** | — | — | — | 15.13 (4.21) | 15.03 (6.73) | .05 (.96) |
| **Disorganization Symptoms** | — | — | — | 5.94 (2.64) | 6.76 (3.12) | .90 (.37) |
| **Reality Distortion Symptoms** | — | — | — | 12.00 (5.91) | 11.85 (7.97) | .07 (.95) |
| **Accuracy** |  |  |  |  |  |  |
| Same Neutral | .91 (.07) | .95 (.05) | 1.61 (.12) | .92 (.06) | .88 (.09) | 1.74 (.089) |
| Same Reward | .93 (.05) | .97 (.03) | 2.22 (.031) | .92 (.06) | .92 (.07) | .12 (.91) |
| Opposite Neutral | .84 (.09) | .88 (.06) | 1.72 (.094) | .81 (.09) | .80 (.10) | .35 (.73) |
| Opposite Reward | .89 (.08) | .91 (.05) | 1.24 (.22) | .86 (.06) | .83 (.10) | 1.11 (.27) |
| **Reaction Time ms** |  |  |  |  |  |  |
| Same Neutral | 556.84 (63.61) | 539.26 (46.70) | .94 (.35) | 551.74 (55.81) | 567.33 (57.53) | .93 (.36) |
| Same Reward | 543.62 (69.03) | 521.79 (39.60) | 1.39 (.17) | 541.24 (55.3) | 554.48 (53.48) | .83 (.41) |
| Opposite Neutral | 602.47 (72.10) | 580.34 (52.06) | 1.04 (.30) | 602.99 (51.89) | 613.56 (65.23) | .58 (.56) |
| Opposite Reward | 588.87 (70.00) | 569.31 (50.83) | .95 (.35) | 599.30 (63.21) | 612.82 (59.01) | .76 (.45) |
| **fMRI Movement** |  |  |  |  |  |  |
| X-Translation mm | .16 (.21) | .17 (.13) | .10 (.92) | .14 (.11) | .17 (.12) | .85 (.40) |
| Y-Translation mm | .17 (.10) | .11 (.05) | 2.79 (.008) | .16 (.10) | .18 (.09) | .85 (.40) |
| Z-Translation mm | .40 (.33) | .37 (.25) | .30 (.77) | .47 (.28) | .70 (.88) | 1.06 (.30) |
| X-Rotation mm | .35 (.35) | .24 (.19) | 1.16 (.25) | .28 (.28) | .34 (.29) | .71 (.48) |
| Y-Rotation mm | .12 (.07) | .13 (.09) | .20 (.84) | .13 (.12) | .14 (.08) | .27 (.79) |
| Z-Rotation mm | .13 (.22) | .12 (.10) | .63 (.92) | .11 (09) | .12 (.09) | .55 (.59) |
| **Activation in ROIs** |  |  |  |  |  |  |
| Dorsal ACC | .33 (.78) | .35 (.99) | .07 (.94) | .46 (.59) | .33 (1.07) | .46 (.65) |
| Right Anterior Insula | .54 (.83) | .80 (.78) | 1.02 (.31) | .21 (.79) | .22 (.90) | .04 (.97) |
| Left VS | .42 (.52) | .63 (.59) | 1.23 (.23) | .32 (.56) | .29 (.99) | .12 (.91) |
| Right VS | .50 (.69) | .68 (.63) | .84 (.40) | .33 (.41) | .41 (.85) | .46 (.65) |

**Supplementary Figure 1***.* Diagram of Incentivized Control Engagement Task (ICE-T) parameters and timing. In "Same" trials, participants were instructed to press the button in the same hand as the matching star (e.g., left hand if the matching probe was on the *left* side of the screen). In "Opposite" trials, participants were instructed to press the button in the opposite hand as the matching star (e.g., left hand if the matching probe was on the *right* side of the screen). Neutral vs. rewarded trials were color-coded, with the color code (red or green) counterbalanced between individuals. Figure from (Smucny et al., 2021).

**
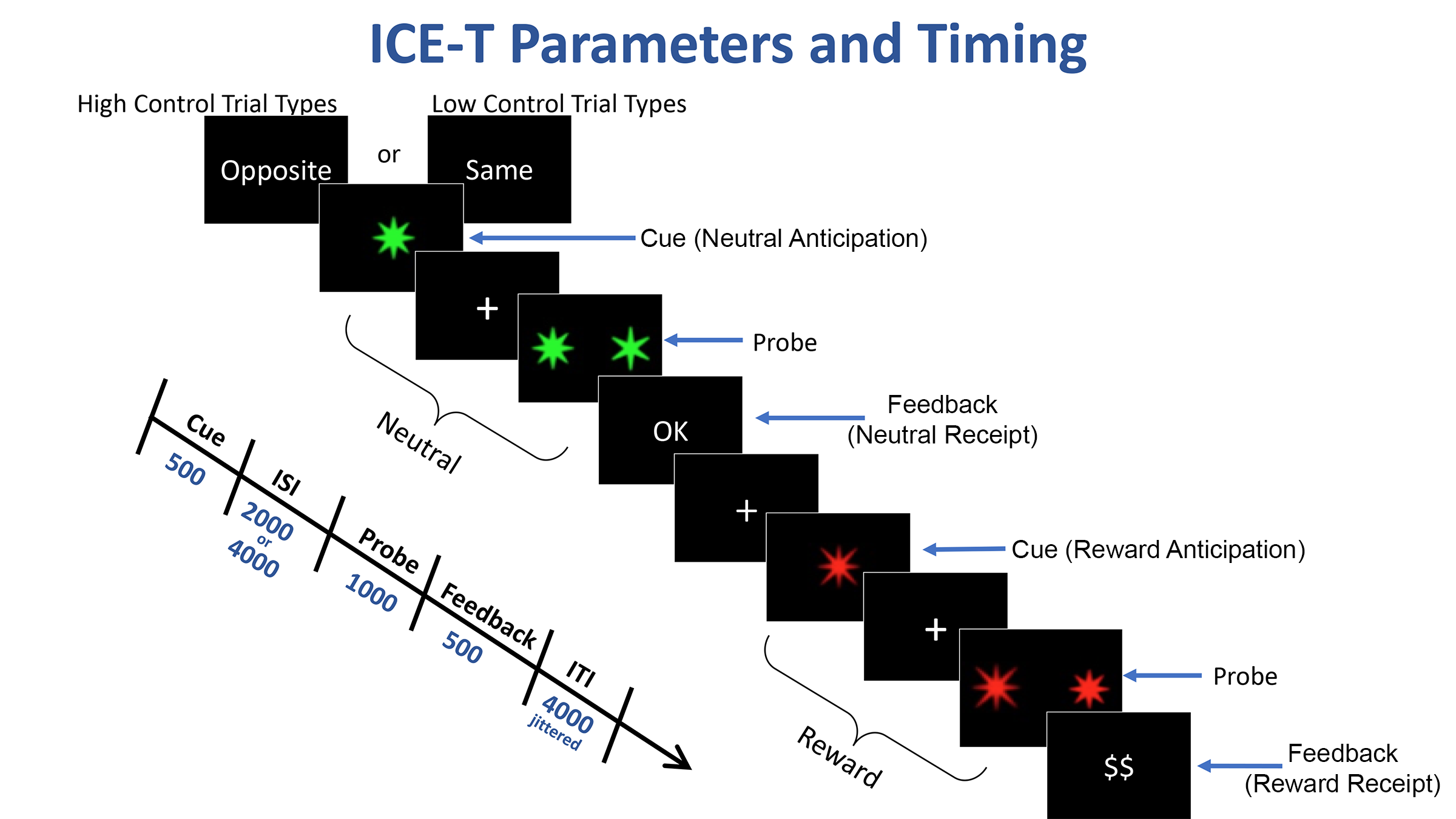
**

**Supplementary Figure 2.** Map showing locations of regions of interest (ROIs). Displayed ventral striatum ROI for the left hemisphere. ROI MNI coordinates were centered at x=2, y=30, z=32 for the dorsal anterior cingulate, x=38, y=20, z=-8 for the right anterior insula, x=-10, y=10, z=-2 for the left ventral striatum, and x=12, y=12, z=-4 for the right ventral striatum.

**
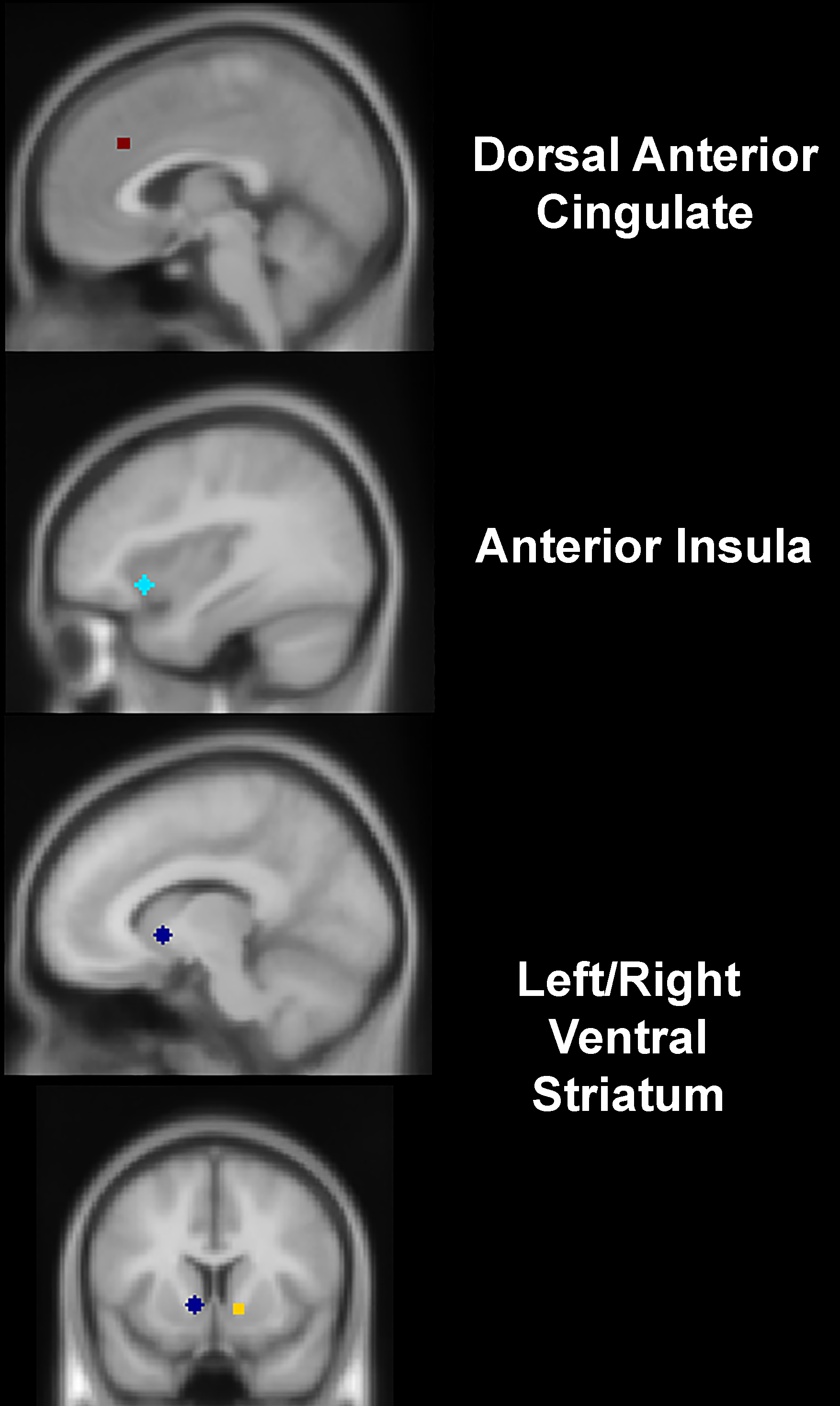
**

**Supplementary References**

Smith, S. M. (2002). Fast robust automated brain extraction. *Hum Brain Mapp, 17*(3), 143-155. doi:10.1002/hbm.10062

Smucny, J., Tully, L. M., Howell, A. M., Lesh, T. A., Johnson, S. L., O'Reilly, R. C., . . . Carter, C. S. (2021). Schizophrenia and bipolar disorder are associated with opposite brain reward anticipation-associated response. *Neuropsychopharmacology, 46*(6), 1152-1160. doi:10.1038/s41386-020-00940-0

Ursu, S., & Carter, C. S. (2005). Outcome representations, counterfactual comparisons and the human orbitofrontal cortex: implications for neuroimaging studies of decision-making. *Brain Research Cognitive Brain Research, 23*(1), 51-60. doi:10.1016/j.cogbrainres.2005.01.004

Ursu, S., Clark, K. A., Stenger, V. A., & Carter, C. S. (2008). Distinguishing expected negative outcomes from preparatory control in the human orbitofrontal cortex. *Brain Research, 1227*, 110-119. doi:10.1016/j.brainres.2008.06.033
